# The challenge of optimizing supports for people living with and beyond cancer: creating proximity between cancer and non-profit community-based providers

**DOI:** 10.1007/s00520-022-07569-3

**Published:** 2023-01-10

**Authors:** Dominique Tremblay, Nassera Touati, Susan Usher, Barbara Gentil, Marie-Josée Courval

**Affiliations:** 1 School of Nursing, Université de Sherbrooke, Longueuil Campus, 150 Place Charles-Le Moyne, Longueuil, Québec J4K 0A8 Canada; 2 Centre de Recherche Charles-Le Moyne, 150 Place Charles-Le Moyne, Longueuil, Québec J4K 0A8 Canada; 3 École nationale d’administration Publique, 4750 Ave Henri-Julien, 5E Étage, Montréal, Québec H2T 3E5 Canada; 4 Centre Intégré de Santé et de Services Sociaux (CISSS) de la Montérégie-Centre, 3120 Boul. Taschereau, Greenfield Park, Québec J4V 2H1 Canada

**Keywords:** Cancer care, Proximity, Community-based organizations, Coordination, Unmet needs

## Abstract

**Purpose:**

Non-profit community-based organizations (CO) remain insufficiently integrated into cancer networks. Drawing on dimensions of proximity, this study explores how and why coordination between cancer teams and COs is established and solidified.

**Methods:**

A descriptive interpretive study is undertaken in Québec (Canada), where a cancer program has long promoted the integration of COs in the cancer trajectory. Semi-directed interviews with providers, managers and people living with and beyond cancer (total *n* = 46) explore the challenges of coordination between cancer and CO providers, along with facilitating or impeding factors. Three main themes related to coordination in cancer networks emerge, which are analyzed by operationalizing the multi-dimensional framework of proximity.

**Results:**

Findings reveal a lack of cognitive proximity, which calls for efforts to both identify patient needs and increase cancer team knowledge and appreciation of CO resources. Organizational proximity refers to systems and rules that facilitate interactions, and we find that referral mechanisms and communication channels are inadequate, with patients often playing a linking role despite barriers. Coordination improves when relational proximity is established between cancer and CO teams, and this can be enhanced by geographic proximity; in one region, COs have a physical presence within the cancer center.

**Conclusion:**

Integrating COs into the cancer network can help meet the spectrum of needs faced by people living with and beyond cancer. This study offers managers and decision-makers insight into how coordination between cancer teams and COs can be supported. Proximity allows the distinct contributions of actors to be considered in context and contributes to understanding the “how” of integrated practice.

## Introduction

Cancer and its treatments generate multiple needs that evolve over time, touching all aspects of life [1–4]. The cancer journey requires care, services and supports that extend beyond specialized cancer teams. Feuerstein and Ganz [5] propose looking to the *Chronic Care Model* (CCM) [6] as a means of optimizing the quality of care throughout the cancer journey, emphasizing the value of complementary community supports to meet needs that fall outside the purview of cancer specialists. Non-profit community-based organizations (hereafter CO) offer a variety of services that can help meet the needs of people living with and beyond cancer [7] as a chronic disease [8]. However, insufficient coordination with specialized cancer teams contributes to unmet needs despite the availability of CO services [1, 2, 7, 9, 10]. “Care coordination is about what happens in the space between providers” [11]. There has been little scholarship on how these separate worlds could better connect, despite findings that people with cancer benefit from using community services [12]. This calls for concerted efforts to understand what happens in the space between providers to coordinate their actions. We draw on proximity dimensions [13, 14] to understand how providers relate to one another and coordinate their unique and complementary contributions in network-based practices.

## Study objectives

This study investigates how and why coordination between cancer teams and COs is established and solidified.

## Background

The dimensions of proximity offer a heuristic to address the “how” and “why” of coordination between actors in integrated cancer networks [15]. Exploring integration through this approach allows the distinct contributions of different actors (health system leaders, cancer team members, COs, people with cancer) to be considered in context. We already know the shortcomings of one-size fits-all solutions to highly contextualized coordination issues [16–18].

Scholars have identified different dimensions of proximity and there is some overlap in how these are defined [13]. Table 1 presents definitions of four of the proximity dimensions [15] especially relevant to understanding the space between specialized cancer teams and COs and how it may be overcome.

**Table 1:** Definition of proximity dimensions

Geographic proximity	Objective (physical) proximity in a given territory [13] and subjective geographic proximity [19] defined as how actors perceive the distance that separates them
Relational proximity	Interactions that enable the development of trust between actors [20]
Cognitive proximity	Shared mental model of a problem or situation and potential solutions [13, 21]
Organizational proximity	Implicit or explicit routines or rules that facilitate interactions between actors; shared systems of beliefs and representations [22]

Geographic proximity is often regarded as the basis for developing other dimensions of proximity, notably the trust and knowledge implied in relational proximity [20]. Cognitive proximity relates to the development of shared mental models that recognize the various distinct contributions to defining a problem and finding actionable solutions [13, 21]. Organizational proximity facilitates coordination of work by increasing the predictability of behaviour and actions [23]. It may be achieved through the development of shared practices or language that can be supported by technology [24]. Some authors consider that organizational proximity also encompasses cognitive, institutional, cultural and social dimensions of proximity [22].

To better understand coordination between cancer teams and COs, we look at how these dimensions of proximity present in the cancer network.

## Study setting

**Fig. 1:**
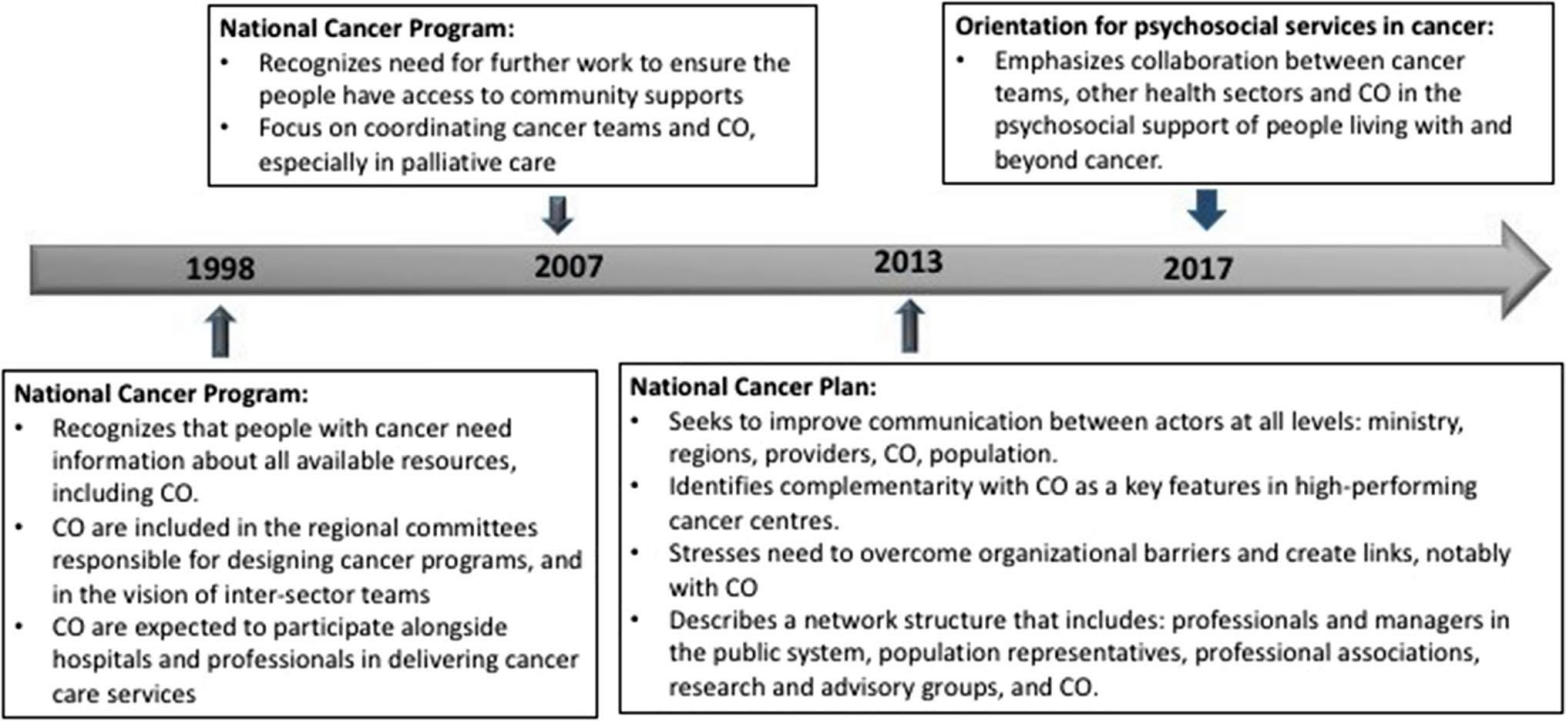
Community organizations in an evolving Québec Cancer Program [26, 30–32]

The creation of proximity between cancer and community-based teams is explored through a case study undertaken in Québec (Canada), where there is a publicly funded health and social service system, and a strong tradition of COs [25]. Figure 1 depicts the timeline of the Québec Cancer Program initiated in 1998, which promotes the contribution of COs as a complement to cancer teams to improve patient experience during the cancer trajectory [26]. Local COs in Québec offer a variety of services, such as accompaniment to appointments, transportation, self-management support, listening, friendly visiting, housing during treatment and meal delivery [27, 28]. Some larger national organizations help people navigate the system and provide referrals to other services offered in the community. The Canadian Cancer Society recognizes more than 4000 COs related to cancer [27, 29]: some offer services that can also be used by the general population, while others offer services more particularly tailored to the needs of people with cancer. The Cancer Plan of 2013–2015 specifies that actors in establishments and in the community will participate in a collective effort towards a common goal of assuring quality care and services and better health outcomes for patients and the population [30]. However, no clear modalities are described for identifying people’s needs and steering them towards the appropriate community-based resources.

## Methods

The study design is Interpretive Description (ID), appropriated to qualitative research that seeks an integrative understanding of a phenomenon of practice interest [33]. ID studies are conducted in the natural setting and value subjective and experiential participant knowledge. They generate results useful to practitioners in applied disciplines.

### Participants and procedure

Study participants (people living with and beyond cancer, providers, and managers in cancer teams and COs) were from two institutions with academic affiliation that provide investigation, acute treatments (chemotherapy, radiation therapy, and immunotherapy) and survivorship services along the cancer journey. These institutions serve a population of nearly 50,000 people distributed across semi urban and urban territories between 1300 and 15,000 km^2^ in size. The type and location of CO services varies, similarly to the whole Québec cancer network. Participants brought different perspectives on the relationship between cancer teams and COs. Selection criteria are described in Table 2. Purposive sampling was used to identify key informants with experience that would contribute to the study objective [33]. Potential informants were contacted by email from an initial list of institutional partners and were recruited using a standard recruitment scenario.

**Table 2:** Participant inclusion criteria

Type of participant	Inclusion criteria
People living with and beyond cancer	A confirmed cancer diagnosis; currently receiving or having received treatment with curative intent, other than strictly palliative end-of-life treatment over the past 12 months
Specialized cancer team clinicians and managers	A professional working for more than a year in cancer care with cancer patients and survivors; a manager working with these professionals, knowledgeable about cancer care and services
CO team providers and managers	A person working in a community-based organization; more than one year experience providing services to people with cancer; a manager of a CO offering care and services, including to people with cancer

### Data collection

Semi-directed interviews (*n* = 46) were conducted with participants by members of the research team who have considerable experience in qualitative studies, along with two Masters’ students. Table 3 presents the breakdown of participants by role. An interview guide was used to promote exchange during the interviews accompanied by a socio-demographic questionnaire. Open-ended questions, adapted to participant’s roles, focused on illustrative cross-boundary interactions between patients, cancer team members and CO workers (e.g., Are you aware of the availability of both hospital and community support resources? If so, how did you learn about them? Thinking about the overall cancer journey, are there any services or resources for which access is facilitated by formal referencing? How do you think awareness of the tools and resources available in the community network could be improved for cancer patients? How do you think we could improve the connections between the specialized cancer teams and community workers? Could you to tell me about a particularly positive experience during transitions between cancer care and community services? A less positive experience?). The various perspectives provided rich description of starting conditions, inputs and core mechanisms more likely to reduce the distance between the health and community sectors. Interviews lasted 40 to 60 min, were audio-recorded and transcribed verbatim.

**Table 3:** Type and sociodemographic characteristics of participant (*N* = 46)

Type of participant	CO team members	Cancer team members	PLWBC^1^
Managers	3	3	
Social workers		4	
Physicians		3	
Nurses	2	3	
Pivot nurses^2^		5	
Community workers	4		
CO services users			19
**Sub-total**	***n* = 9**	***n* = 18**	***n* = 19**
Characteristics of participant n (%^3^)			
Gender			
Male	4 (44)	3 (17)	3 (16)
Female	5 (56)	14 (74)	15 (79)
Age, (mean, range)	44 (28–58)	44 (30–61)	59 (35–78)
Employment status			
Full time	4 (44)	15 (83)	
Part time	4 (44)	3 (17)	3 (16)
On sick leave			5 (26)
Social welfare			1 (5)
Retired			9 (47)
Education level^4^			
Low education			6 (32)
Middle education	2 (22)		6 (32)
High education	5 (56)	18 (100)	7 (37)
Primary cancer type^5^			
Breast			12 (63)
Colon			3 (16)
Lung			2 (11)
Malignant melanoma			1 (5)
Hodgkin lymphoma			1 (5)
Treatment phase^6^			
Current treatment			15 (79)
Post treatment			4 (21)
Living status			
With partner			8 (42)
With partner + child			2 (11)
Single			6 (32)

^1^PLWBC: people living with and beyond cancer recruited in ambulatory clinics in participant sites.

^2^Pivot nurse: also called nurse coordinator or nurse navigator in oncology responsible for patient evaluation, intervention, education, coordination, and professional navigation

^3^Percentage not always equal to 100% due to missing value or after rounding

^4^Education level: low (primary or secondary school); middle level (professional or collegial); high (university)

^5, 6^Self reported by PLWBC.

### Data analysis

**Fig. 2:**
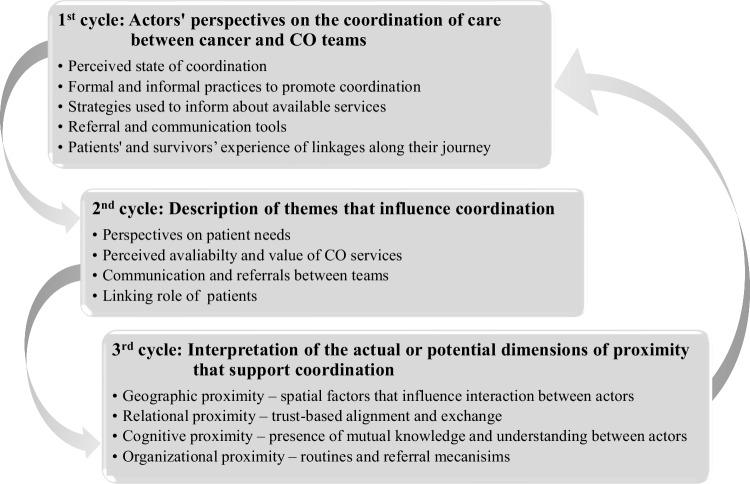
Three coding cycles

Thematic analysis was undertaken through three coding cycles (Fig. 2) to understand proximity dimensions that contribute to coordination between specialized cancer teams and teams in COs [33, 34]. A first cycle coded raw data according to the interview guide, which was developed to document the type of relationships that existed between cancer and community-based team members, the context in which these links took place, and processes in place to coordinate care between teams. People living with and beyond cancer were asked to describe their experience navigating between cancer and CO teams. These raw data were attributed codes reflecting the nature of participant statements. A second cycle was undertaken to describe main themes that influenced the relationship between teams. Three main themes emerged: *perspectives on patient needs and on the availability and value of community-based services; communication and referrals between teams; and the role of people with cancer in navigating cancer and community-based teams*. A third coding cycle interpreted the actual or potential dimensions of proximity generated by these efforts to support coordination.

### Quality criteria

A logbook was kept as an audit trail and reflexive tool to record thoughts on data and methodological process and relate them to elements of the conceptual framework; insights were discussed to ensure that participant propositions were accurately understood [35]. Members of the research team coded the interview data independently in each cycle, then came together to discuss divergences and achieve consensus. Further data from the field (internal documents such as tools used for coordination, chart reviews, meeting minutes, follow-up notes, along with team meeting observation) allowed for data triangulation to enhance credibility [35].

## Results

Coordination between cancer and community teams remains uneven and weak overall, despite the ambitions stated in the national cancer plan. Our findings provide deeper understanding of coordination from the perspective of cancer teams, CO and people living with and beyond cancer, and point to barriers as well as efforts that appear promising to overcome them. Perspectives of cancer team members reveal an incomplete understanding of how people’s needs can be met by services available from CO. There is a lack of mechanisms or tools in place to facilitate and encourage communication between teams. People living with and beyond cancer are left to assume a linking role, but face hurdles in doing so. We then rally dimensions of proximity to describe elements that appear fundamental to generating improvements in these areas. The benefit of proximity is that it helps understand a variety of specific efforts that might work to coordinate actions in different contexts and circumstances. Results integrate findings around proximity dimensions within the three themes that emerged in the second stage of analysis.

### Perspectives on patient needs and the availability and value of community-based services

*Cognitive proximity* denotes a common understanding of problems and potential solutions that enables actors to recognize each other’s contributions. Our findings point to the insufficiency of mechanisms in place in cancer teams at either of the study sites to identify people’s needs in order to steer them to the appropriate community resources.

More than [the cancer team] saying "contact us if there's a problem", they should take the lead, and tell people: "Look, you may still need help, but it may not be medical help; here are some resources you should contact". They don't do that here. – (manager, CO)

Cancer teams have screening tools available for evaluating symptoms and distress. However, their communication to providers outside the team remains sensitive and at the provider’s discretion and does not necessarily lead to referral to a CO that could meet these needs. Nor is the evaluation consistently used in communication with patients to help them equip themselves to cope with the impacts of cancer on various aspects of their life: anticipate needs, find resources, prepare for self-management and recognize the alarm signals that should trigger a request for assistance. Information exchange and feedback loops between cancer teams, patients and CO are rare.

They fill in the screening for distress tool, but don't seem to do anything with it [in terms of referral to CO services]. In theory, you complete a tool because it will be used for something, but here there's no next step other than when the patient says they're happy and all is well. – (manager, CO)

When referrals are made to COs, it is often without having first identified a person’s specific needs and ascertained the appropriateness of a given community service. This reduces the referral’s coherence with the perceived needs of people living with and beyond cancer: they may not be ready or favorably disposed to receiving the service at the time the referral is made; or they may face disappointment as the service to which they are referred does not meet their needs as expected. Cognitive proximity suffers through a lack of common vision of the problem between patient and cancer team, and a lack of common vision of CO service offerings between cancer teams, community-based teams and patients. Tools such as screening for distress and needs assessments are one component, but must be accompanied by conversations and specific knowledge of available service options.

### Effective communication and referrals between cancer and community-based teams

Cancer and CO teams lack *organizational proximity*, or common routines and referral mechanisms, that would facilitate the exchange of information and knowledge to ensure that people living with and beyond cancer are referred to appropriate community services. Organizational proximity is impeded by the uneven availability or use of referral mechanisms and communication channels.

No, the hospital didn't give me anything, nor did the oncologist refer me to community services. Nothing. – (person living with cancer)

There should really be automatic referral mechanisms, and a chance for us to talk. You know there's no communication channel with the cancer network for COs; it doesn't exist at the moment and it prevents them [cancer teams] from understanding our reality. – (community worker, CO)

Contrary to what is prescribed in Québec’s Cancer Plan, there are no formal bidirectional service corridors in place. Results show few signs that cancer teams recognize the complementarity of services provided by COs. Cancer teams are not always confident that services in the community will be available to people in their region.

COs meet certain needs, but services are not available everywhere. Transport, for example, works well in some regions, but not in others. I know that pivot nurses make a lot of referrals, but service availability is uneven. – (cancer team member)

As well, accurate up-to-date information is difficult to maintain as there is considerable variation between regions in the availability of community services, and COs may lack the resources to communicate regularly with and receive feedback from cancer teams. Some COs produce promotional material and cancer survivors often find out about services via newspapers or pamphlets in their local community, often in pharmacies. However, without activation from the cancer team, it becomes a matter of chance whether or not a person comes across the right information at the right time.

If no link is made before they're discharged from hospital, it's left to chance whether they find out that (name of the CO) exists, despite the fact that it's well known and promoted. The COs all do a lot of promotion in their areas, but it becomes the luck of the draw whether the patient will happen upon a pamphlet; that's not a referral. – (manager, CO)

One cancer team participant describes how use of a referral form allows them to have a conversation with the patient prior to discharge from hospital about their needs, and have them sign consent to have the referral passed on to the CO, which can then call the patient directly. However, this practice is not widespread. Other efforts seek to create organizational proximity by formalizing information pathways and referral practices. On one study site, the cancer team is in the process of integrating a referral form to community services into its standard processes. The form will include 20 services available locally for people with cancer, in areas such as accompaniment, transport, psychological support, healthy living, stress management, help with activities of daily living, support groups, wigs, help with adapting to life with cancer, with return to work, etc. In another region, a large non-profit organization plays the role of first contact with patients for all the other community resources. This facilitates initial referrals by cancer teams.

So, what we committed to doing was to make the initial contact with each resource, and only once we're sure that contact has been established, put the person in touch with the resource, because we don't want them to have to go through the trouble, explain their situation all over again, etc, etc. We want to make the connection as easy as possible. – (manager, CO)

*Relational proximity* is seen to compensate in some instances, for the lack of *organizational proximity*. Cancer team participants mention a few specific community services, notably in palliative care, with which they have established relational proximity and practice informal referrals. Referrals often result from somewhat random initiatives of the personnel in one or the other setting. Some pivot nurses report developing good relationships with COs and can orient patients to the services they need. Once the patient is in touch with a CO for a particular service such as transport, the CO has an opening to mention other services they provide or direct people to other local COs.

### The role of people with cancer in navigating cancer and community-based teams

The creation of *relational proximity* often relies on people living with cancer, who take steps themselves to identify community services that seem able to meet their needs. However, in interviews, some people living with and beyond cancer describe their efforts to contact COs as frustrating, because they cannot connect with an actual person; instead they are meant to leave their information and wait for a return call. They also describe needing their oncologist’s signature to request some community services, and oncologists are not always receptive to such requests. People living with and beyond cancer report finding it hard to undertake the effort to contact COs due to fatigue, emphasizing that first contacts need to be easy and, ideally, initiated by the CO.

I'd appreciate them telling me about it, because, you know, you're tired, you don't feel good about yourself, you don't feel like searching. It would be great if it was delivered on a platter, you know, easy. If they gave you the information, not just on paper, but had someone call you, because you know, sometimes it (the information) registers and sometimes it doesn't. Sometimes, you just don't feel like reading. – (person living with cancer)

The role of people living with and beyond cancer in bridging cancer teams and COs is made more difficult when cancer teams lack faith in COs’ capacity to meet the needs identified in or expressed by people with cancer. As well, cancer teams are not always confident that COs will act on referrals once they are made.

### Supporting coordination

Dimensions of proximity operate interdependently [15]. Geographic proximity appears, in our findings, to provide a basis for the development of other dimensions of proximity, and have potential to generate improvements on all three factors that affect coordination. In one region, a representative of local COs has a dedicated space within the cancer centre, where they can hear people’s needs and provide them information about relevant community services. This colocation appears promising for generating cognitive proximity around the needs of people with cancer and the value of community services, and facilitating organizational and relational proximity by improving communication between cancer and community teams and enabling patients to play a linking role.

I don't know how many referrals they (the community organizations) get, but if they're getting more, it's because of the new regional cancer centre, where they have more visibility, and the cancer and palliative and end-of-life care departments are committed to giving them more prominence. – (cancer team member)

I appreciate the pamphlet listing community services they give you in oncology when you're waiting to see the oncologist; there's a sheet with all the workshops. – (person living with cancer)

Despite some promising strategies, we find a pervasive lack of coordination between cancer and CO teams that impedes access to community services for people living with and beyond cancer. Cancer team knowledge of community services and understanding of how they contribute to meeting people’s needs remain underdeveloped. People living with and beyond cancer are left to assume much responsibility for determining their needs, identifying appropriate services and making contact. However, they face barriers: fatigue, feeling others deserve services more, difficulty finding out about services and establishing contact.

In interviews, actors identify a number of efforts to create or heighten coordination that operate by generating cognitive, organizational, geographic, and relational proximity. These proximity dimensions enable us to ask: does this action create new systems or practices that reduce barriers to coordination? Does it help actors develop a common vision of the problem? Does it contribute to trust in the contributions of each party (i.e. that the cancer team will refer appropriately/that the CO will meet the patient’s need). The particular effort may differ from one context to the next, but produce similar effects.

## Discussion

In Québec, the Cancer Plan provides a strong central mandate to coordinate the actions of various teams to ensure that the needs of people living with and beyond cancer are met [30]. Paying attention to dimensions of proximity appears as a promising complement to structural efforts to increase network coordination. These subtler contributions to coordinate care and services help to mature network integration by developing common ideas around the nature of needs and ways to meet them, and by facilitating communication between actors that develops mutual understanding, trust and interdependency [36].

Proximity dimensions allow us to understand how the space between actors can be altered to promote integration by facilitating connections between healthcare providers, community supports and people living with and beyond cancer. The creation of such linkages remains imperfect in chronic disease generally, and the complexities of cancer add further layers of difficulty. Leeuw [37] emphasizes the need for conceptual tools to understand complex problems. The present study adds specific insights that may help integrate COs more fully into cancer networks. A first is recognition that cognitive proximity around what needs can be met by COs is not yet present, and this impedes the development of organizational and relational proximity to facilitate integration. Cancer teams and COs are as water and oil, coexisting but separate, with just a few sporadic bubbles making their way (at great effort) across. This represents a first work path for cancer systems. A second is that people living with and beyond cancer are vital linking agents, but do not always recognize their own needs, obtain knowledge about available services, or perceive themselves as eligible for community services [38]. This last emphasizes an important role for cancer teams in normalizing use of community services. The creation of cognitive proximity about needs and the potential of different resources to meet them must also extend to, and work through, people living with and beyond cancer [39]. Detection tools do not, on their own, generate this cognitive proximity; to support coordination, they must be accompanied by patient partnership and feedback loops that are embedded in daily practice.

The creation of organizational proximity appears as a condition for coordination, calling on efforts from healthcare establishments to increase direct interaction between cancer teams and COs. Such efforts may, however, face resistance from COs: integration into cancer networks can raise the specter of loss of independence and instrumentalization to fill gaps left by disinvestment in the public sector [40]. Proximity offers a means of addressing integration while supporting the autonomy and recognizing the distinct contribution of different participants [15].

The chronic care model was developed to guide health system improvement and implies “linkages at different, multiple, and optimally coordinated levels” [41p. 22]. However, studies of interactions across levels and sectors remain rare. Strange et al. recognize, in studies of non-cancer multi-level initiatives, that policy changes (to reorient funding), organizational adaptations (such as new office systems), and practice-level changes (coordination of referrals) all play a role in meeting patient needs, and that “simple but complementary interventions at different levels may have greater effect than intensive interventions at a single level” [41p. 26].

A survey of adults living with and beyond cancer in 10 Canadian provinces [8, 42] finds that 59% of those who express practical concerns, such as getting to and from appointments, do not receive help. Fully 88% of those who express emotional concerns do not receive help. Improving coordination between cancer teams and COs is one route to better meeting the range of needs expressed by people living with and beyond cancer. Experience in Québec and elsewhere reveals that cancer network integration requires more than network structures. Efforts to create proximity fall under the less tangible elements of integration that produce a common system of reference [43] or shared mindset [15, 44]

## Strengths and limitations

Data triangulation, meaning comparison of data from independent sources and data from interviews with key actors who bring different perspectives — cancer teams, community-based teams and people living with and beyond cancer — increases the credibility of findings and contributes to internal validity [35]. While factors such as the education level of people with cancer were not addressed, the varied characteristics of participants contribute to the credibility of the study.

We cannot, however, claim that the sample is representative of all actors, which limits the transferability of findings [35]. For example, in Québec, professional navigation is part of the role of the pivot nurse in oncology (also called nurse coordinator, nurse navigator) to reduce barriers to care along the cancer trajectory [45]. Although pivot nurses participated in the study, none of the study participants addressed the role of lay patient navigators assist people find resources from COs [46]. Optimizing lay patient navigators overcome organizational fragmentation and barriers to formal referral in favor of more informed activated patients, and prepared, proactive cancer and CO teams as suggested in the CCM. Data are collected in two regions of Québec, the people living with cancer recruited are largely women with breast cancer, the investigation and palliative phases of the cancer trajectory are not represented. These characteristics must be considered in assessing the transferability of findings, along with features of the Québec context that may be more or less present in other jurisdictions.

## Conclusion and future research

Improving coordination between cancer and CO teams requires action within the health system, but must also address fragmentation in the community sector. The institutional features of COs warrant further attention to understand how different models of funding and regulation impact on coordination with cancer teams, and on people’s perceived eligibility to use their services. The expansion of social economy models alongside more traditional philanthropic models warrants attention from this perspective, as does the influence of larger chapter-based models. The challenge of assuring complementarity and coordination while maintaining independence appears central and could be explored through the organizational dimension of proximity [39]. This study addresses an important gap in our understanding of cancer care coordination, and the larger problem of fragmented care and support for people living with and beyond cancer. Although there is no magic bullet for creating a real continuum of care, the framework of proximity with is spatial and non-spatial dimensions may offer a new tool to shift from team-based care to a multi-team cancer system.

## Data Availability

Data are held by the research team and may be available from the corresponding author upon reasonable request and with relevant approvals.

## Abbreviations

CCM: Chronic care model
CO: Non-profit community-based organization
ID: Interpretive description
PLWBC: People living with and beyond cancer
